# Supplementary material for: The interactome of intact mitochondria by cross-linking mass spectrometry provides evidence for coexisting respiratory supercomplexes
Source: Mol Cell Proteomics. 2017 Dec 8;17(2):216–32. doi: 10.1074/mcp.RA117.000470 (PMC5795388; doi:10.1074/mcp.RA117.000470)
Supplement: Supplemental Data [file supp_RA117.000470_133922_0_supp_23978_fzffwf.zip › spectra_annotation/mito_DR_spectra_annotation/255-1-4-1-9-1.pdf]

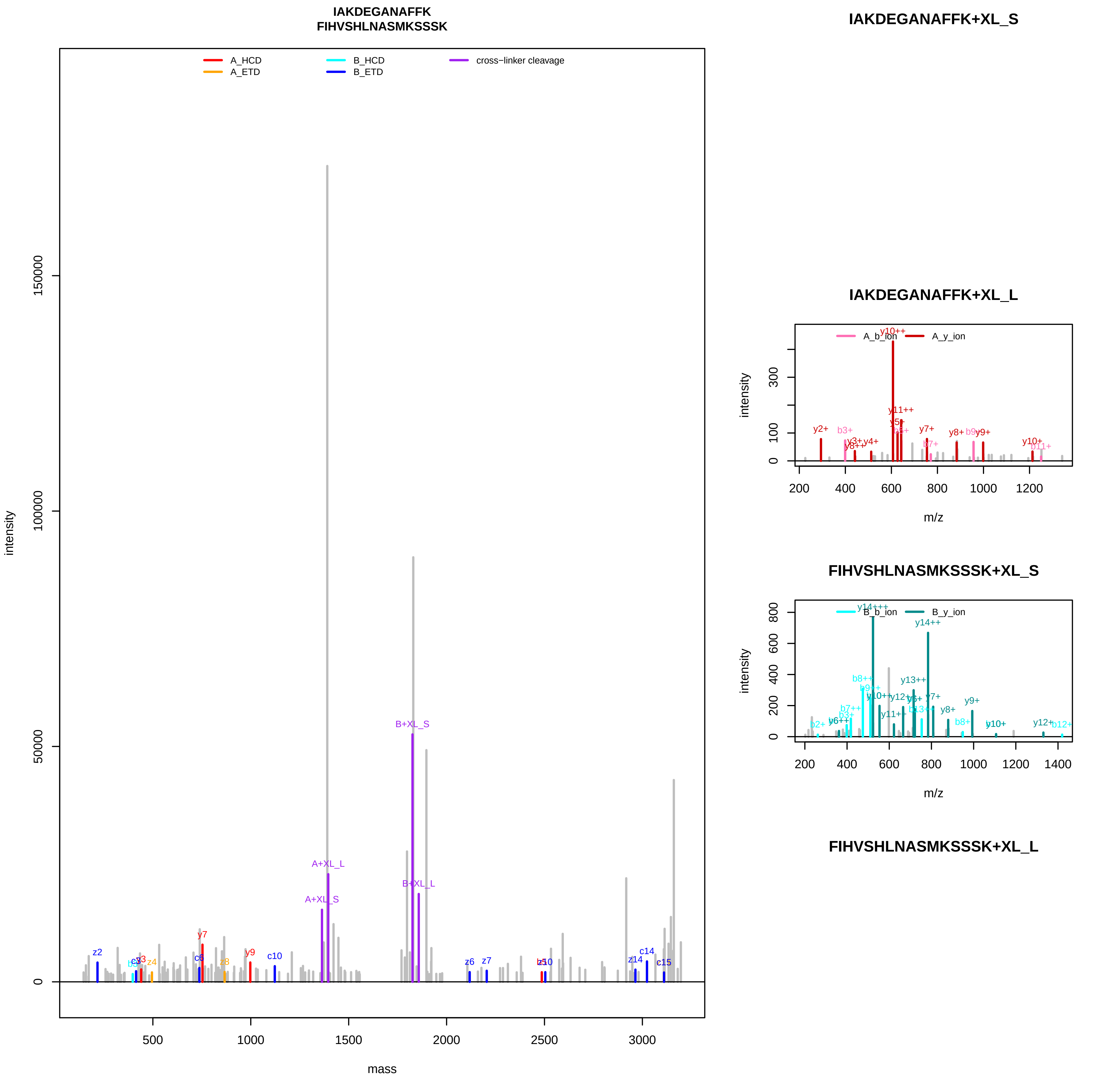
IAKDEGANAFFK
FIHVSHLNASMKSSSK
A_HCD
A_ETD
B_HCD
B_ETD
cross−linker cleavage
intensity
mass
0
50000
100000
150000
500
1000
1500
2000
2500
3000
A+XL_L
A+XL_S
B+XL_S
B+XL_L
z2
c3
z3
z4
c6
y7
z8
y9
c10
z6
z7
z10
z14
c14
c15
IAKDEGANAFFK+XL_S
IAKDEGANAFFK+XL_L
A_b_ion
A_y_ion
y10++
y11++
y2+
b3+
y3+
y4+
y7+
y8+
y9+
y10+
b11+
b7+
m/z
0
100
300
200
400
600
800
1000
1200
FIHVSHLNASMKSSSK+XL_S
B_b_ion
B_y_ion
y14+++
y14++
y13++
b8++
y10++
y12++
y7+
y8+
y9+
y11++
b7++
b3+
b2+
y6++
b8+
y10+
y12+
b12+
1400
FIHVSHLNASMKSSSK+XL_L
